# Artificial Intelligence-Based Secured Power Grid Protocol for Smart City

**DOI:** 10.3390/s23198016

**Published:** 2023-09-22

**Authors:** Adel Sulaiman, Bharathiraja Nagu, Gaganpreet Kaur, Pradeepa Karuppaiah, Hani Alshahrani, Mana Saleh Al Reshan, Sultan AlYami, Asadullah Shaikh

**Affiliations:** 1 Department of Computer Science, College of Computer Science, and Information Systems, Najran University, Najran 61441, Saudi Arabia; aaalsulaiman@nu.edu.sa (A.S.); hmalshahrani@nu.edu.sa (H.A.); 2 Chitkara University Institute of Engineering and Technology, Chitkara University, Rajpura 140401, Punjab, India; bharathiraja@chitkara.edu.in; 3 Department of CSE, St. Michael College of Engineering and Technology, Kalayarkoil, Sivaganga 630551, Tamil Nadu, India; pradeepa.k94@gmail.com; 4 Department of Information Systems, College of Computer Science, and Information Systems, Najran University, Najran 61441, Saudi Arabia; msalreshan@nu.edu.sa (M.S.A.R.); asshaikh@nu.edu.sa (A.S.)

**Keywords:** smart city, cyber Security, power grids, recurrent neural network, edge-cloud-assisted, secured grid

## Abstract

Due to the modern power system’s rapid development, more scattered smart grid components are securely linked into the power system by encircling a wide electrical power network with the underpinning communication system. By enabling a wide range of applications, such as distributed energy management, system state forecasting, and cyberattack security, these components generate vast amounts of data that automate and improve the efficiency of the smart grid. Due to traditional computer technologies’ inability to handle the massive amount of data that smart grid systems generate, AI-based alternatives have received a lot of interest. Long Short-Term Memory (LSTM) and recurrent Neural Networks (RNN) will be specifically developed in this study to address this issue by incorporating the adaptively time-developing energy system’s attributes to enhance the model of the dynamic properties of contemporary Smart Grid (SG) that are impacted by Revised Encoding Scheme (RES) or system reconfiguration to differentiate LSTM changes & real-time threats. More specifically, we provide a federated instructional strategy for consumer sharing of power data to Power Grid (PG) that is supported by edge clouds, protects consumer privacy, and is communication-efficient. They then design two optimization problems for Energy Data Owners (EDO) and energy service operations, as well as a local information assessment method in Federated Learning (FL) by taking non-independent and identically distributed (IID) effects into consideration. The test results revealed that LSTM had a longer training duration, four hidden levels, and higher training loss than other models. The provided method works incredibly well in several situations to identify FDIA. The suggested approach may successfully induce EDOs to employ high-quality local models, increase the payout of the ESP, and decrease task latencies, according to extensive simulations, which are the last points. According to the verification results, every assault sample could be effectively recognized utilizing the current detection methods and the LSTM RNN-based structure created by Smart.

## 1. Introduction

A Smart City (SC) uses Information Technology (IT), Artificial Intelligence (AI), and the Internet of Things (IoT) to gather, evaluate, and combine critical information about fundamental systems of current cities to increase infrastructure and durability [1]. An SC would be a concept that makes a reality by calculating a sizable quantity of data acquired by detectors, cameras & energy techniques to manage resources, offer services, and address problems in every living [2]. SC provides prompt, efficient, and intelligent fulfilment of needs related to city services, public security, daily living, and commercial and industrial activity [3]. All systems are based on power or energy [4]. As a result, it serves as one of the solid foundations for implementing the SC concept.

Modern conventional electrical grids are becoming intelligent grids. Conventional grids lose power during generation, distribution, and transmission because of their unidirectional communication [5,6]. The introduction of SG addresses traditional grid issues and has free bi-directional connectivity. Control systems, information systems, and devices for bi-directional communication were all included in developing safety data sheets (SDS). SDSs have developed into an essential source of safety data for both business and research.

On the other hand, when the SDS contains inaccurate information, terrible mishaps may happen [7]. Modern phasor networks are a component of this sophisticated equipment. Managers could assess grid stability thanks to these sensing devices [8].

Additionally, SG has controlled feeder switches for rerouting electricity in the event of a grid failure, an innovative digital system that gives users better information and spontaneous feedback, recognizes problems, and batteries with the extra power to fulfil a future user need [9]. An electric grid’s rapid transition creates chances and obstacles to improving the current power distribution system [10]. Electricity prediction was crucial for the smart grid’s efficient control and reduction of operational expenses. Future trends are provided via load and price prediction.

Intelligent algorithms are becoming more feasible because of recent advancements in data technology & the availability of large and different amounts of information to PG [11]. Machine Learning (ML) approaches have benefited from their inherent generalization competency compared to traditional computational techniques. An ability to control power and connection networks where real-time information on energy, cost, power quality, usage, and other factors are connected to the system’s components presents one of the main obstacles to employing SG [12]. Furthermore, the focus is on creating intelligent systems that could make decisions even in unclear situations utilizing ML techniques. The balance between energy generation and consumption controls the grid’s resilience [13]. With more power being produced and decentralized, power flow has expanded to include both directions. These improvements require an intelligent control center to balance production and energy consumption [14]. ML techniques evaluate the strong correlation between the grid stability parameters and input units for heterogeneous consumers [15].

Nevertheless, convolutional neural networks (CNNs) can accurately represent the structural locality from the feature space. Because they employ local frequency area pooling, they also lessen translational variance and handle disruptions and minor shifts in the feature space. By taking advantage of previous speech signal information, they may take advantage of the long-term relationships between speech frames. The ASR systems’ performance suffers because CNNs cannot handle the stress of semi-clean data. On the other hand, recurrent neural networks (RNNs) provide improved recognition accuracy by collecting lengthy contexts, particularly for noise-resistant applications. However, the vanishing and expanding gradient issue constrained RNNs’ capacity to learn temporal dependencies.

Long short-term memory (LSTM), which manages information flow through a unique unit known as a memory block, was developed to address these issues. Target delays about features occur because LSTM-RNNs are sensitive to static data. Acoustic modelling has significantly benefited from using deep neural networks (DNNs). Convolutional neural networks (CNNs) are an improved form of deep neural networks (DNNs), with a 4–12% relative improvement in word error rate (WER) over DNNs. CNNs are more effective at recognizing speech when spectral fluctuations and local correlations exist in the speech stream.

The texts must be legible by machines to use digital tools for text research. Major modern languages like English have freely accessible digital corpora of texts that can be large and constructed from digital texts. Signs may be layered and positioned on top of one another using RES. New sign operators are part of RES, although higher-level processing is needed. The lifetime of the encoding and its font-independent semantics are also points in favour of RES. The current study shows that RES is likewise considerably more advantageous. The relative placement of scanned signs may be directly translated to the appropriate encoding primitives using a widely established parsing method. Using artificial intelligence (AI), text mining extracts helpful information from text. It transforms unstructured input into structured data using NLP, an acronym for natural language processing. Machine learning (ML) algorithms and analysis both require this. All of this is raw, unorganized data. It cannot be searched, no patterns can be found, and no keywords can be obtained without automatic text mining. However, when you begin text mining and organizing this data, it benefits your business. You may review client reviews and the terms they frequently use to decide how to alter communication or cost. What is the subject that comes up most in your emails? The list continues on and on.

### 1.1. Research Gap

Due to the modern power system’s rapid development, more scattered smart grid components are securely linked into the power system by encircling a wide electrical power network with the underpinning communication system. By enabling a wide range of applications, such as distributed energy management, system state forecasting, and cyberattack security, these components generate vast amounts of data that automate and improve the efficiency of the smart grid. Due to traditional computer technologies’ inability to handle the massive amount of data that smart grid systems generate, AI-based alternatives have received a lot of interest. Long Short-Term Memory (LSTM) and recurrent Neural Networks (RNN) will be specifically developed in this study to address this issue by incorporating the adaptively time-developing energy system’s attributes to enhance the model of the dynamic properties of contemporary Smart Grid (SG) that are impacted by Revised Encoding Scheme (RES) or system reconfiguration to differentiate LSTM changes & real-time threats. More specifically, we provide a federated instructional strategy for consumer sharing of power data to Power Grid (PG) that is supported by edge clouds, protects consumer privacy, and is communication-efficient.

### 1.2. Authors Contribution

Long Short-Term Memory (LSTM) and recurrent Neural Networks (RNN) will be specifically developed in this study to address this issue by incorporating the adaptively time-developing energy system’s attributes to enhance the model of the dynamic properties of contemporary Smart Grid (SG) that are impacted by Revised Encoding Scheme (RES) or system reconfiguration to differentiate LSTM changes & real-time threats. More specifically, we provide a federated instructional strategy for consumer sharing of power data to Power Grid (PG) that is supported by edge clouds, protects consumer privacy, and is communication-efficient. In this study, they develop a detection technique to identify the attacked bus and manage the ambiguities introduced by combining RES and scenarios. The time-identifying energy system at-tributes was merged into a separate category learning approach to overcome the abovementioned concerns and identify FDIA to actual time. For example, our study is the first to create a new real-time detection approach based on DL approaches to accurately and sufficiently reflect the dynamic characteristics of current power systems. Deep Learning (DL) methods are well established. The implementation of DL approaches has significantly enhanced methodology in most disciplines of research where DL has led. Several neural networks, such as deep auto-encoders, Convolutional Neural Networks (CNN), and recurrent neural networks (RNNs), have been developed in recent years.

### 1.3. Manuscript Organization

The rest of the paper is structured as follows. We consolidate the related works in Section 2. Section 3 details the materials and methods. Section 4 discusses the experimental results for performing the CNN method’s predictions on the MNIST databases. The discussion is provided in Section 5. We present the conclusion and future works in Section 6.

## 2. Related Works

The determination of the reliability of the smart grid would be a fascinating area of study because this knowledge could be beneficial in identifying the variables that contribute to its volatility and, consequently, in selecting configurations that maintain the grid’s reliability even when some of its components exhibit irregularities [16]. The main task in creating an SC is gathering and analyzing essential data. SG could use ML to make critical judgments about peak load, changes in customer needs, and grid resilience [17,18]. This has aided in forecasting the grid’s strength under the consumers’ constantly shifting demands. Researchers could comprehensively understand the gathered data with ML methods [19]. This has aided in transforming the smart grid into a strong one, which would help prevent failure scenarios.

IoT devices use communication and network technology to continuously create information in SC and deliver it to network services [20]. Costly data centers and equipment are needed for massive information collection and processing in cloud services. Process data at the gadget end is a clear answer to this issue. Edge computing describes these kinds of computing devices that exist at the farthest limits of a network [21,22]. Edge computing IoT smart grid technologies save network utilization and processing time through power management and scheduling. Smartphones are used by informed citizens to interact with cloud services [23]. With the reduction in data size brought on by preprocessing, mobile edge computing techniques can provide continuous latency-free services without considering the location. Only pertinent data were transmitted after this treatment removed any redundant information [24]. Researchers proposed the green survivable virtual network embedding and used a wireless mesh network for cooperative edge computing. Devices in a sensor network could use their downtime for network operations and prepare device information.

A DL method identifies key false data injection attack (FDIA) traits and then applies the learned attributes to FDIA detection using historical data [25]. In generative adversarial networks, autoencoders were explicitly used to identify measurements subject to FDIAs by detecting divergence irregular and standard measurements [26]. This creates a method that effectively detects deviations from false data injection (FDI) to look at temporally continuous predicted variables by combining deep neural network techniques & wavelet transform developed. It is neither economically advantageous nor difficult practically to deliver energy through transmission lines at locations where the main grid and electricity usage are remote, such as remote islands and segregated communication stops. Under these circumstances, the best action is to deliver electricity using a microgrid (MG) incorporating renewable energy sources in an islanding mode. The two major components of smart island (SI) security are physical and cyber security. The capacity of an SI to continue operating normally in the face of significant interruptions is represented by physical security. The security of computer systems and communication networks that help the SI function is called cyber security.

Due to the widespread use of information technology, cyber security has recently become a danger to smart cities and SI systems. Additionally, because of the intricate interconnection of the physical and cyber systems, cyber security flaws may pose a danger to the SI’s physical security. Cyberattacks can undermine or completely disrupt the control systems supporting electric power networks. It has long been assumed that cyber-attacks cannot threaten industrial system security. The traditional method of managing MG activities involves a central supervisory controller and data collecting, suitable for high-level operations, including global optimization and agent duty. This controller recommends an effective coalition of crucial subsystems required for reliable performance. Generally speaking, the current detectors use the computed probability to identify future attacks that cause considerable deviations from the reference distribution. They first approximate the underlying distribution of previous observations [27]. The proposed methodology was compared qualitatively to the pertinent literature, utilizing four components for each method.

Recently, deep learning techniques have been proposed to sort the detector characteristics into layers to capture the higher-order statistical structure of the complex data. Deep belief networks, produced using a collection of restricted Boltzmann machines (RBMs), are one of the fundamental deep learning approaches frequently utilized. To deploy neural networks effectively, selecting the right variables is essential. Potential attackers view the MGs as obvious targets, typically controlled via voltage and current measurements. The neural network that serves as the foundation for the deep learning algorithm has numerous hidden layers in addition to its input and output levels. It can simulate complex non-linear relevance between numerous types of variables. Layer by layer, unsupervised learning is used to acquire these network parameters, and supervised learning is used to fine-tune them. Deep learning patterns lead to increasingly complex features at higher output layers, and by varying the input, the complex details will remain constant.

AI algorithms can analyze data from various sensors, including smart meters, power substations, and grid sensors, to identify abnormal patterns or potential threats. These threats could include cyber-attacks, physical intrusions, or equipment failures. AI can continuously monitor the grid and flag any suspicious activities for further investigation. AI techniques such as machine learning can establish baseline behaviour models for the power grid. By continuously monitoring the grid’s real-time data, AI algorithms can compare the current state with the established models and identify deviations or anomalies. It also enables early detection of potential security breaches or operational irregularities.

AI can leverage historical data and real-time information to forecast potential grid vulnerabilities or security risks. By analyzing patterns and trends, AI algorithms can predict potential failures, cyber threats, or even power demand fluctuations. These predictions enable proactive measures to mitigate risks and enhance grid security. AI-powered cybersecurity systems can detect and respond to cyber-attacks on the power grid. AI algorithms can analyze network traffic, identify malicious activities, and swiftly respond to prevent further damage. Additionally, AI can aid in identifying vulnerabilities in the grid’s digital infrastructure and recommend security patches and updates.

AI can automate the response to security incidents by providing real-time recommendations to grid operators or initiating automated actions. For example, AI systems can isolate compromised components, reroute power flows, or trigger backup systems to maintain an uninterrupted power supply. This reduces response time and enhances the grid’s resilience. In a smart city, multiple AI systems can be deployed across various infrastructure components, including power generation, distribution, and consumer endpoints. These AI systems can collaborate and share information to provide a holistic view of the power grid’s security. Collaborative AI networks enable comprehensive threat analysis and response coordination.

AI algorithms can continuously learn from new data and adapt to emerging threats. By leveraging reinforcement learning techniques, AI systems can improve their ability to detect and respond to evolving security risks. Regular updates and integration of AI models ensure that the secured power grid protocol remains practical and up-to-date. It’s important to note that implementing such a secured power grid protocol requires a collaborative effort among various stakeholders, including power utilities, technology providers, and regulatory bodies. Additionally, privacy concerns and data protection should be addressed to ensure the responsible use of AI in securing the power grid of a smart city.

While implementing a secured power grid protocol for a smart city using artificial intelligence (AI) brings numerous benefits, it also presents several challenges. Some key challenges need to be addressed: AI-powered systems require access to significant data from various sources to analyze and secure the power grid effectively. Ensuring the security and privacy of this data is critical. Safeguarding sensitive information from unauthorized access, maintaining data integrity, and complying with relevant privacy regulations pose challenges that must be addressed to gain public trust.

While AI can enhance grid security, it also introduces new cybersecurity risks. AI systems can be vulnerable to attacks, and adversaries may attempt to manipulate or disrupt AI algorithms to compromise the power grid’s security. Ensuring the robustness and resilience of AI systems against cyber threats is a significant challenge. Adversarial attacks involve intentionally manipulating input data to deceive AI algorithms and cause erroneous or malicious behaviour. In a secured power grid protocol, adversaries may attempt to exploit vulnerabilities in AI models to bypass security measures or generate false alarms. Developing AI models that are resistant to adversarial attacks is a complex challenge.

The accuracy and reliability of data are crucial for the effectiveness of AI algorithms. However, power grid data can be noisy, incomplete, or inconsistent. Additionally, data availability might vary across different locations or infrastructure components. Ensuring the quality and availability of data for AI-based security systems is a significant challenge that requires robust data collection and preprocessing mechanisms. Smart cities generate vast amounts of data from diverse sources and processing this data in real-time poses scalability challenges for AI algorithms. Power grids require timely responses to security threats, and AI systems must be capable of analyzing and acting upon data in real time. Developing scalable AI architectures that can handle the volume, velocity, and variety of data in an innovative city environment is a complex task.

The energy transition-revolution paradigm introduces a fresh perspective on interaction models to intelligently manage the energy and data transferred among all players in the whole power system with a firm focus on sustainability, resilience, cybersecurity, and privacy. Demand-side management (DSM), a combination of software and hardware models and data analytics capabilities, is an essential facilitator of the energy transaction and the transfer from fossil to renewable energy sources. In addition to demand response capabilities, the DSM manages the grid infrastructure, reducing customer pain and optimizing grid stability in light of environmental obligations [28]. Any network system loses efficiency, dependability, and resilience when cybersecurity is neglected. The Connected Electric Vehicle (CEV) as a mode of transportation in cities is becoming more and more effective and able to respond to citizen and environmental expectations, improving the quality of citizens’ lives. This is due to the comprehensive incorporation of the Information, Communication, and Technology capabilities. However, this advancement in CEV technology makes it more susceptible to cyber-attacks, posing a major risk to citizens. As a result, they have a more significant potential to harm society and people physically, resulting in unforeseen financial losses [29].

The huge adoption of electric vehicles (EVs) and renewable energy sources (RESs), particularly wind power, is required to achieve carbon neutrality by 2050 and reduce reliance on fossil fuels. To operate, schedule, maintain, and trade energy in the modern power system, an effective and trustworthy energy management system (EMS) is necessary due to the integration of a significant amount of intermittent wind power into the grid as well as the variability of the load on the demand side [30]. Implementing a secured power grid protocol involves integrating AI systems with existing infrastructure and technologies. Ensuring interoperability between different components, such as smart meters, substations, and control systems, is challenging. Integration requires standardization of protocols, data formats, and communication interfaces to enable seamless collaboration between AI systems and existing infrastructure. AI-powered systems must adhere to ethical principles and legal frameworks. Decision-making algorithms should be transparent, explainable, and accountable to ensure fairness and avoid biases. Addressing ethical concerns, such as job displacement due to automation or unintended consequences of AI systems, is essential to build public acceptance and trust in the secured power grid protocol.

Implementing AI-powered security systems can involve substantial costs, including infrastructure, hardware, software, and skilled personnel. Smart cities may face resource constraints regarding budget allocation and expertise. Balancing the costs and benefits of implementing AI-based solutions and identifying sustainable funding models pose significant challenges. Addressing these challenges requires collaboration among stakeholders, including power utilities, technology providers, government agencies, and researchers. Ongoing research, development, and continuous improvement of AI algorithms and protocols are necessary to overcome these challenges and build secure and resilient power grids in smart cities.

## 3. Materials and Methods

Comparisons are made between how well the centralized Artificial Intelligence IoT (AIoT) models, FL, and MNIST databases perform when the CNN method is used to make predictions. The generated data produced a test set and a training set at random to accomplish the goal. An FL framework with edge-cloud interaction is provided to assess energy data in SG while ensuring privacy and increasing communication efficiency. The conceptual framework was also utilized to develop a local storage assessment technique for cost modelling and two optimization methods for ESPs and EDOs while considering the non-IID distribution of heterogeneous consumers. In the standard cloud-based method in FL, global/local model changes were quickly transmitted between the cloud and EDOs, ignoring integrators. Long Short-Term Memory (LSTM) and Recurrent Neural Networks (RNN) will be specifically developed in this study to address this issue by integrating the adaptively time-developing energy system’s attributes to enhance the model of the dynamic properties of contemporary Smart Grid (SG) that are impacted by Revised Encoding Scheme (RES) or system reconfiguration to differentiate LSTM changes & real-time threats. More specifically, we provide a federated instructional strategy for consumer sharing of power data to Power Grid (PG) that is supported by edge clouds, protects consumer privacy, and is communication-efficient. They then construct two optimization concerns of Energy Data Owners (EDO) and energy service operations, as well as a local information assessment mechanism in Federated Learning (FL), by taking into account non-independent and identically distributed (IID) effects. The test results revealed that Model 3 reproduced the detailed behaviour of the average training loss excellently, and the power systems were adequate. LSTM features a more extended training session and more training loss with four hidden levels. The proposed method performs well in detecting FDIA in a range of situations. Finally, extensive simulations have shown that the suggested technique may induce EDOs to employ high-quality local models, increase ESP payout, and minimize task latencies.

### 3.1. Cyberattack

**Figure 1:**
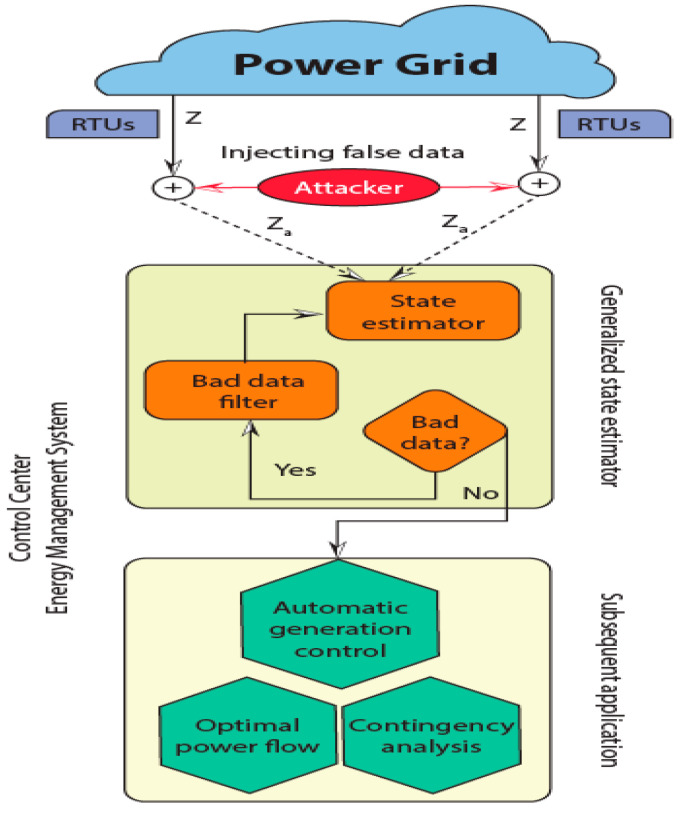
SG cyber-attack on the power system.

The attack is depicted in Figure 1. The energy required grows exponentially with the rising usage of technology and innovations. The rapid expansion of communication infrastructure in smart grids has resulted in new cybersecurity challenges in physical power systems. Traditional approaches to dealing with cyber-attacks on power systems mainly include separating the cyber realm from the physical domain. As a result, it is critical to have a unified cyber and physical power infrastructure. The cyber-physical power system (CPPS) was created to address these concerns. The basic physical power system is strongly connected with the cyber system of the CPPS. CPPS aims to effectively and reliably monitor and regulate smart grids. Power transmission, generation, distribution, use of power, supervisory control and data acquisition (SCADA), and use of electricity are some of the stages that make up the CPPS. Because these phases of smart grids are vulnerable to cyberattacks, compiling, examining, and keeping track of the various cyberattack techniques on CPPS is crucial. When run on the altered measurement data z_a_, the SC would produce the incorrect system state bxa = bx + c. The measurement residual to FDIA the assailant might introduce random error.

(1) $$z_{a}=\mathrm{Hx}+a+e=Z_{a}$$

### 3.2. LSTM-RNN in Modern Power Systems

A feedforward neural network with internal memory expands a recurrent neural network. Because it uses the same function for each data input and depends on the results of the previous input’s calculation, RNNs are recurrent. A copy of the output is created after it is produced and sent back into the recurrent network. It considers the current input and the outcome learned from the previous input. RNNs can process input sequences by utilizing their internal state (memory) in their internal processing. As a result, they can be applied to projects like speech recognition or connected, unsegmented handwriting recognition. The inputs to other neural networks are all unrelated to one another. However, with an RNN, every input is linked to every other.

Long Short-Term Memory (LSTM) networks, which enable higher memory retention for prior information, are created by adjusting recurrent neural networks. Here, the vanishing gradient problem of the RNN is resolved. Applications of LSTM include the categorization, processing, and prediction of time series with ambiguous time delays. Back-propagation is used to train the model. The input gate decides which input value should modify the memory. Find out what data needs to be deleted from the block using the forget gate. The sigmoid function makes that determination. The output is determined by using the block’s memory and input.

Due to feedback connections in the buried level, RNNs could create transient relations between the most current and previous states. On the other hand, the gathered power system measurements are continuous quantities selected to a timestamp and might be understood as sequence information arranged in the period domain. Moreover, in future grids, the energy response to time t + t would be impacted by the topology difference at time t. RNNs would be an excellent option for capturing the dynamic character of SG as a result [31]. Typically, the divergent and algebraic Equations (2) and (3) could also be used to describe the dynamic features of SG:

(2) $$\hat{i}=F\left(i,k,R,H\right)$$

(3) $$j=G\left(i,k,R,H\right)$$

The forward computation procedure could be formally represented as follows to demonstrate the interconnection of elements to the hidden layer created by LSTM:

(4) $$h\left(t\right)=f\left(h\left(t-1\right),I\left(t\right),W,b\right)$$

Semi-explicit Dynamic Algebraic Expressions (DAE) systems fall under this category. The first half f of the equations specifies a distinct direction for x for each solution of the second half g of the equation. However, the direction for y is arbitrary. However, only some points (x, y, t) solve g. The attribute differential is determined by the variables in x and the first half of the equations. The algebraic variables or equations of the system are the components of y and the second half of g of the equations. [The word algebraic in the context of DAEs refers to the absence of derivatives and has nothing to do with (abstract) algebra.] A DAE may be solved in two steps: first, look for beginning values that are consistent, and then, compute a trajectory. The derivatives of certain DAE’s component functions must frequently be considered to arrive at consistent beginning values. The differentiation index is the derivative’s highest order required for this operation. Where I represent the hidden layer outcome variable for the LSTM at time *t*(*t*), the hidden layer state vector for the LSTM at weight matrix and bias vector and time t is represented by *h*(*t*), are each represented to W and b, correspondingly. Regarding mathematical structure, the hidden layer LSTM Equation (4) is virtually identical to the separate version. Therefore, the hidden layer generated by LSTM blocks may be capable of defining complex networks like mathematics summaries of the state vector.

Arithmetically, the developed hybrid neural networks can be expressed as:

(5) $$h\left(t\right)=f\left(h\left(t-1\right),I\left(t\right),W_{2},b_{2}\right)$$

(6) $$O\left(t\right)=g\left(h\left(t\right),I\left(t\right),W_{f},b_{f}\right)$$

where $W_{2}$, $b_{2}$, $W_{f}$, and $b_{f}$ denote LSTM bias vector, hidden layers, and $W_{f}$, $b_{f}$ denote the FC hidden layer weight matrix and the output vector of FC layers as indicated by the notation O(t).

### 3.3. Modeling the Dynamic Behavior of Power Systems

Data from the database are moved to the top hidden units of the input layer. The samples’ non-linear connections are modeled using the LSTM hidden layer [32]. Remote teams were required for classification the more complicated the relations were. After receiving data from the final hidden layer, the FC layer calculates the sample’s likelihood of each category. The output layer receives the possibilities from the final FC layer and identifies the instances [33]. In addition, various factors, including the size of energy systems, the proposed network architecture difference, and the RES adoption, affect the number of hidden units of FC & LSTM levels. Furthermore, experiments that include the effectiveness and computation load of LSTM RNNs are often used to determine the number of remote units for LSTM and FC levels [34].

### 3.4. Proposed Framework Structure

**Figure 2:**
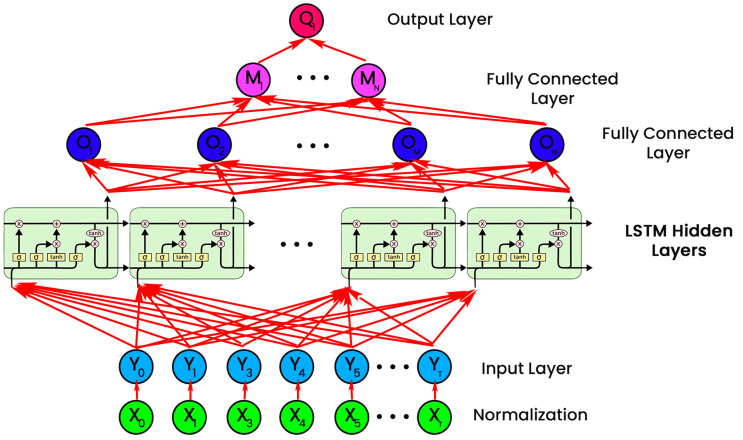
The LSTM network’s intended form of a power system.

**Figure 3:**
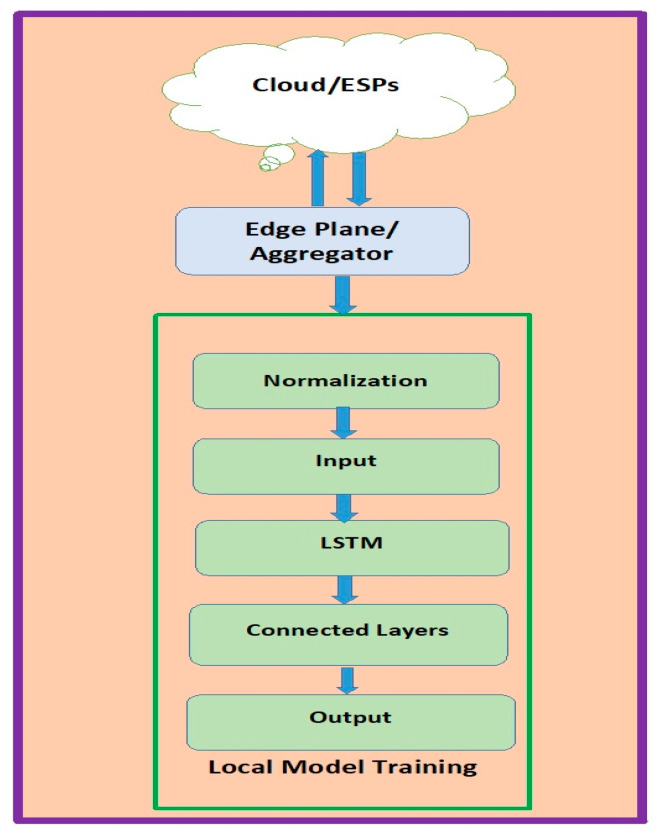
Proposed Research Methodology.

Figure 2 depicts the final design of the LSTM network configured to identify all three types of assaults mentioned. This framework can successfully identify all three forms of attacks and will be detailed in the experimental findings. In other words, the adequately designed LSTM can simulate the dynamic behaviour of modern power systems. Figure 3 depicts the proposed research methodology of work followed.

#### Federated Learning Model

To obtain an accurate STLF forecast for increased power delivery efficiency, FL and short-term load forecasting (STLF) must gather huge quality power information from households as the associated power data in the Extended Data Output (EDO) are constrained.

(7) $$L_{x}\left({⊖}_{y,k}\right)=\frac{1}{S_{x,y,k}}\sum_{\sigma -1}^{S_{x,y,k}}f\left(i_{a};{⊖}_{y,k}\right)$$

The edge-cloud integrated FL paradigm of SG is depicted in Figure 3. It contains a cloud, numerous geographically dispersed integrators, ESPs, and many EDOs. The following steps must be completed to the nth GTE (n = 0, 1, 2...) to solve the federated learning formula under edge-cloud integrated SG: Task publication and model initialization are followed by local updating, edge aggregation, global aggregation, and learning completion. In complicated and dispersed contexts, like those seen in SGs, edge computing may not be able to satisfy some developing requirements. As a result, the necessity of bringing AI to the network’s edge has drawn much attention as a practical method for maximizing the advantages of edge computing. Therefore, edge intelligence (EI) has been created by merging these concepts (edge computing and AI). IoT devices’ computing speed has significantly increased, which is one of the advantages that EI allows. Another benefit of EI is that utilizing dispersed edge resources on the network to increase the energy efficiency of various AI applications successfully minimizes computational dependency on the Internet cloud.

Each Energy Data Owner (EDO) would own SC or smart homes and a collection of heterogeneous electricity usage generated by smart meters, household appliances, electric vehicles, solar panels, etc. Let Si stand for the owner’s power database. The achieved method predicted less the total payment to associated EDOs & the network or the payoff operation of the ESP kth task is:

(8) $$\pi_{y,k}\left(P_{y,k}\right)=\mu v_{\alpha }{∆}_{y,k}-\left(1-\mu \right)\left(\sum_{x\epsilon I_{y,k}}P_{x,y,k}\varphi_{x,y,k}+\lambda_{c}1_{y,k}^{comp}\right)$$

In addition, current approaches and cryptography-based safe-gathering solutions are being used to reduce implicit privacy loss across integrators and the cloud. It could also be used in our system. The proposed method can particularly defend the system against the following assaults, including the Low-quality local method update assault and free-riding assault.

## 4. Results

### 4.1. Numerical Study

**Figure 4:**
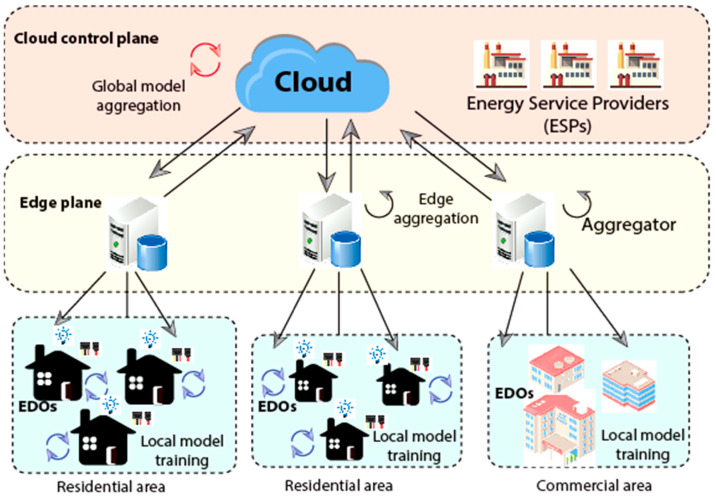
Integration of the cloud and the edge.

**Figure 5:**
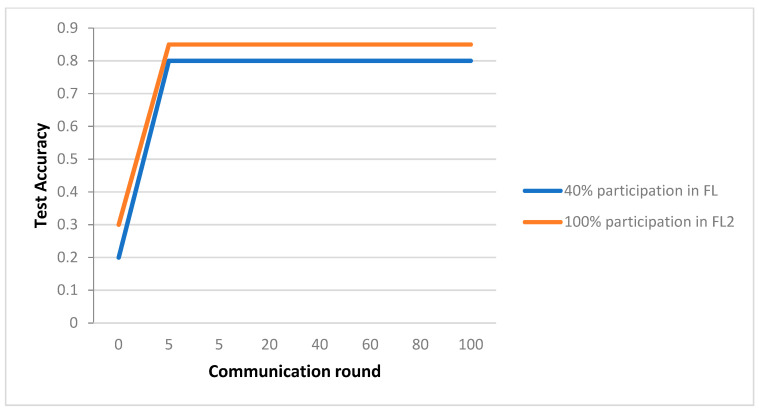
CNN prediction performance compared to MNIST.

The performance of the CNN method’s predictions on the MNIST databases, the FL, and centralized Artificial Intelligence IoT (AIoT) models to various amounts of engagement and communication rounds are compared in Figure 4. As seen in Figure 5, the global model developed using the FL framework could achieve coverage after roughly 30 communication rounds. Additionally, compared to the centralized AIoT strategy’s predictive performance of 99.78%, the FL & AIoT solution could reach 95.8% precision to greater participation in the 100th connection cycle. In Figure 5, the global model developed using the FL framework could achieve coverage after roughly 30 communication rounds. Additionally, compared to the centralized AIoT strategy’s predictive performance of 99.78%, our FL & AIoT solution could reach 95.8% accuracy under full participation in the 100th connection cycle.

**Figure 6:**
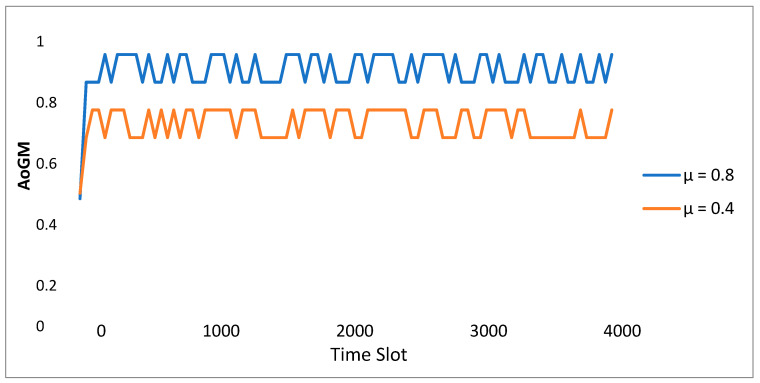
Development of the ESP’s AIoT.

**Figure 7:**
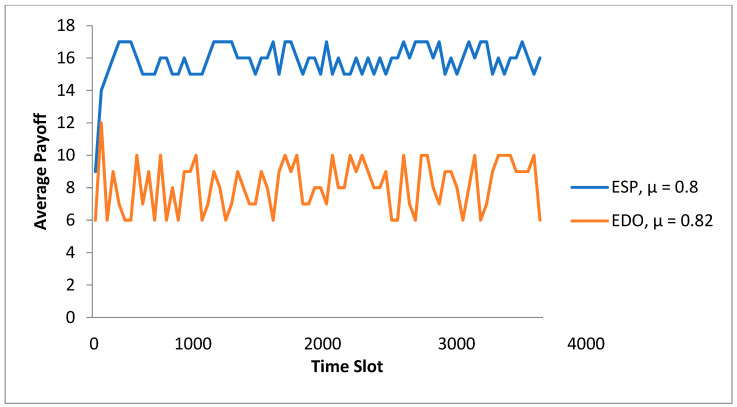
The average payment of ESPs and EDOs over time.

In Figure 6, with the ESP type set to = 0.8, the total payment of the ESP & EDO in the DQN is shown as the period slot varies. The subsidies range from 0.4 to 1.0 kWh, depending on where the utility grid’s pricing adopts the feed-in tariff for distributed PV energy. The internal prices of the various ESRs determined by ESP are displayed in the transmission-distribution pricing. In Figure 6, the ESP plans to reduce the payout to improve the future high reward after noticing the excellent method performance of EDOs driven to the IHPs. EDO tends to reduce the payoff of pursuing higher long-term rewards. When there are between one and ten tasks, the two methods’ combined communication delays are shown in Figure 7. The global/local model modifications were immediately communicated between the cloud and EDOs in the typical cloud-based strategy in FL, disregarding integrators. Figure 7 shows how our suggestion for edge-cloud collaboration could help decrease the communication delay given a variety of job counts. By enhancing network connectivity and capacity, intermediaries in the edge layer help mitigate the effects of unpredictable and intermittent wireless connections across users and the internet. The aggregators could work together network FL through edge aggregation activities. As a result, our edge-cloud collaboration architecture may achieve greater communication efficiency.

### 4.2. Simulation Results

The proposed LSTM RNN-based FDI detector’s effectiveness was verified by calculating the F_1_ score from the confusion matrix. This is how F_1_ is calculated.

(9) $$F_{1}=\left(\frac{2\times P_{r}\times R_{e}}{P_{r}+R_{e\;}}\right)$$

where R_e_ represents recall, and P_r_ represents precision, and these two metrics are determined as follows:

(10) $$P_{r}=\left(\frac{True\;Positive}{True\;Positive+False\;Positive}\right)\;R_{e}=\left(\frac{True\;Positive}{True\;Positive+False\;Negative}\right)$$

**Figure 8:**
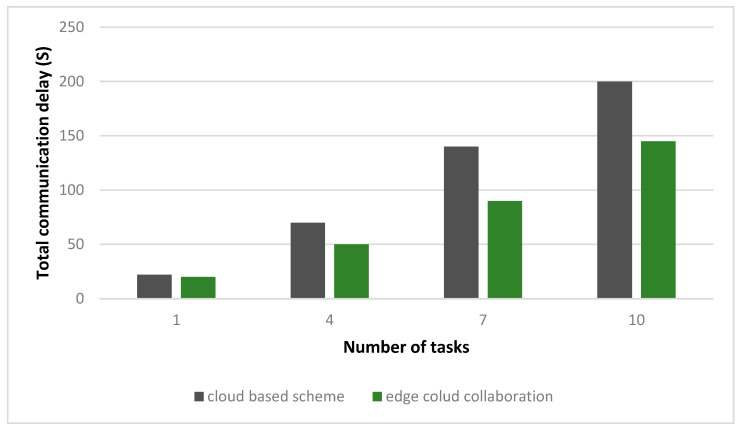
The overall communication delay to the number of assignments in the two systems.

True Positive would be the proportion of accurately discovered regular observations, True Negative is the quantity of accurately found assault measures, False Positive is the number of incorrectly observed assaults, and False Negative missed assaults. Any measurement designated as usual is average, according to the F_1_ value 1, while any sample assigned as assaulted was controlled. A subset of the case study simulation data assesses these structures. According to test results, Model 3 was perfect for simulating the complex behaviour of average training loss, and the power systems were tolerable. As a result, in this study scenario, a real-time assault identification method is built using Model 3. The LSTM could not converge for the first two models, and the training loss was significant on average. With four concealed levels, LSTM has shown a longer training time & more considerable training loss. A more complicated LSTM framework would also raise the likelihood of overfitting. Figure 8 displays the four models’ training loss.

Moved to the input layer, the top hidden units are database-related data. The LSTM hidden layer simulates the nonlinear connections in the examples. The more complex the relationships were to classify, the more remote teams were needed. After obtaining data from the final hidden layer, the FC layer determines the sample’s likelihood for each category. The output layer takes the potentials from the top FC layer and finds the instances. Various variables, including the size of energy systems, projected network architecture variations, and the adoption of RES, also influence the number of hidden units of FC & LSTM levels. In addition, tests examining the efficiency and computational burden of LSTM RNNs are frequently used to estimate the required number of remote units for LSTM and FC levels.

The chosen Framework has been used to evaluate many variables to determine the ideal parameters. A test set & a training set were randomly created from the generated data to achieve this goal. It is essential to remember that there are more options, like batch size, learning rate, optimization method, and stages. However, they primarily have an impact on how long the practice takes. The outcomes of verifying the Smart designed LSTM RNN-based structure and the current detection methods in the first case study are presented in Table 1. Practically every approach was able to identify every assault sample accurately.

**Figure 9:**
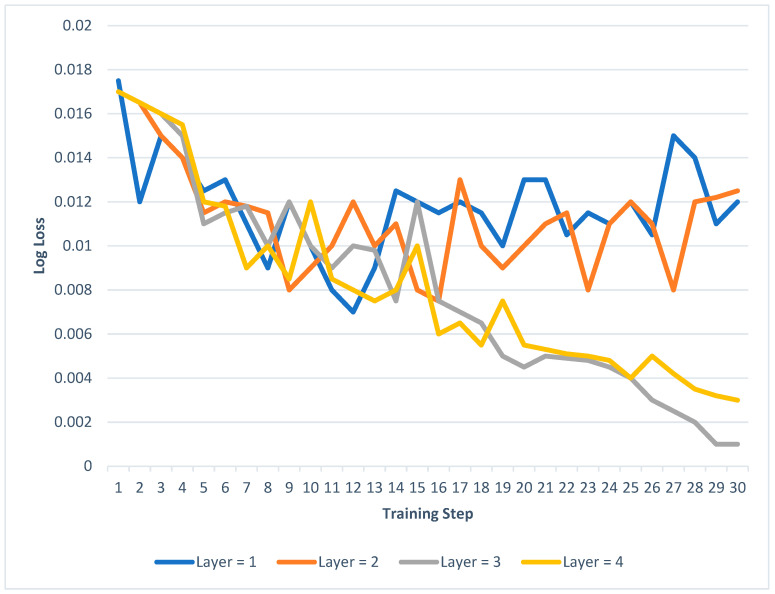
The learning loss of LSTM RNNs with 1 to 4 hidden units.

**Figure 10:**
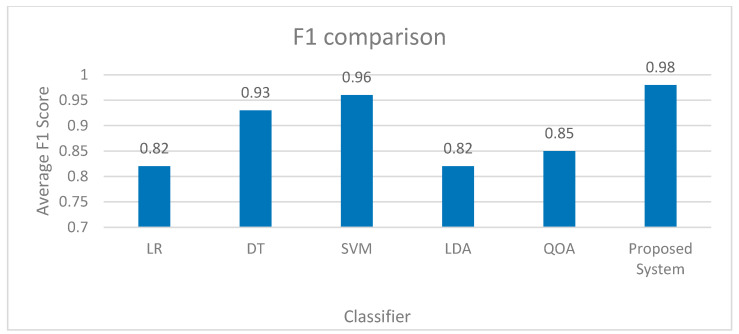
Comparison of Proposed and Existing System.

A condensed representation of the simulation results is shown in Figure 9. As one can see, the proposed methodology performs excellently in diagnosing FDIA in various situations, whereas the current sensors endure a significant decrease in accuracy. It is robust to modern power grid disturbances. The comparison of proposed and existing systems is shown in Figure 10.

## 5. Discussions

The operation of smart cities is fundamentally dependent on the modern power infrastructure. However, the security of the urban power infrastructure is severely hampered by high-effect, low-likelihood catastrophic events. The resilience of urban power grids has become a priority for a contemporary smart city due to the growing attention to these dangers. A robust electrical grid can withstand interruptions, adapt to them, and quickly recover from them. It has four traits: anticipatory, absorbing, adapting, and recovering. Urban intelligence levels have significantly increased due to the fast development and widespread use of technologies for communication and information. The notion of a “smart city” is also created by various smart urban infrastructures, gaining much attention from academics and businesses. The major goal of a smart city is to promote urban economic growth while simultaneously enhancing the quality of life for its inhabitants. Safe, sustainable, effective, low-carbon, and resilient are qualities that make a smart city successful, and this assumes that the city has intelligence like that of humans. It is crucial to have a resilient, safer, lower-carbon, and greener urban power infrastructure.

In this study, the authors develop a detection strategy to locate the attacked bus and handle the ambiguities brought about by combining RES and eventualities. The time-identifying energy system attributes were merged into a separate category learning approach to overcome the abovementioned concerns and identify FDIA to actual time. For example, our study is the first to properly develop a unique actual time detection strategy leveraging DL approaches to reflect the dynamic characteristics of contemporary power systems accurately and effectively. The techniques for deep learning (DL) are well established. The approaches in most of the disciplines of research that DL has led have been significantly enhanced by applying DL techniques. Convolutional neural networks (CNN), recurrent neural networks (RNNs), and deep auto-encoders are only a few of the neural networks that have recently been developed.

In this research, we provide a federated learning-based AIoT method for private energy data sharing in collaborative smart grids with edge clouds that is both efficient and safe. In particular, we present a federated learning framework that is communication-efficient and privacy-preserving for user energy data sharing in smart grids. Then, considering non-IID impacts, we develop two optimization problems for EDOs and energy service providers (ESPs) and build a local data assessment method in federated learning. Additionally, a two-layer deep reinforcement learning-based incentive algorithm is created to encourage EDO participation and high-quality model contribution due to the need for more understanding of multi-dimensional user private information in real-world settings. According to extensive simulation findings, the suggested system can effectively encourage EDOs to exchange high-quality local model updates and increase communication efficiency.

Aggregating flexibly distributed resources safely and reliably has been a critical component of power grid resilience due to the intricate interaction of many uncertainties in urban power grids. Future aggregation technologies are needed for contemporary urban distribution grids to monitor and harness the enormous flexibility of resources. Cyber-physical systems (CPS), which incorporate cyber, physical, and social systems, are expanded into cyber-physical-social systems (CPSS). A smart city will have more urban infrastructure operators due to CPSS’s promotion of information and energy resources from one dimension to three. Several cutting-edge technologies are being used to increase the resilience of the urban electricity system. To improve distribution network resilience against future cyberattacks, for instance, a soft open points-assisted technique is suggested. A self-organizing map-based strategy to allocate resources and dispatch operations is suggested, which can mitigate some drawbacks of systems that assign weights subjectively. Peer-to-peer (P2P) energy trading methods are used in distribution networks to control the volatility of renewable energy output and lower the risk of uncertainty from severe occurrences.

Battery storage stations and wind-hydrogen storage can improve distribution system resilience by increasing battery capacity, improving safety technologies, and lowering prices. Furthermore, mobile energy storage can provide localized assistance to critical loads during an extreme event. Compared to permanent storage, its mobility enables operational flexibility to accommodate geographically scattered loads. By combining a diverse set of cross-disciplinary and cutting-edge technologies, resilience improvement solutions for urban power grids in smart cities become increasingly adaptable and practical against extreme occurrences.

## 6. Conclusions and Future Work

As a result, a search is made for a clear understanding of the distinctions between an assault on data security and a natural transition to the current power grid. This constraint was extensively addressed to deal with this paper’s high volatility and unpredictability using an LSTM RNN-based architecture. In both the training and evaluation sets, the created detector model could accurately depict the dynamic nature of future power grids. An FL framework with edge-cloud integration was offered to evaluate energy data in SG while protecting privacy and maximizing communication efficiency. The conceptual methodology was also used to build a local storage evaluation method for cost modeling and two optimization methods for ESPs and EDOs while considering the non-IID distribution of heterogeneous customers. Finally, in-depth simulations have shown that the suggested approach may effectively drive EDOs to great-quality local models, enhance the payout of the ESP, and shorten task latencies. Since the modern grid is still in its infancy, there is still much room for improvement and application of this idea. It is currently unclear how much more research will be needed in smart grids to realize this idea. Still, recent advancements in the field, including those involving smart meters, demand-side management systems, self-healing, and big data, are encouraging. We hope the CPPS will provide fresh insight into planning and creating a smarter grid.

## Figures and Tables

**Table 1:** Method Assessments.

Performance Metrics	Accuracy	Precision	Recall	F-Score	AUC (ROC)
Logistic Regression (LR)	0.82	0.81	0.79	0.82	0.9
Decision Tree (DT)	0.92	0.9	0.9	0.91	0.9
Support Vector Machine (SVM)	0.96	0.96	0.95	0.96	0.98
Linear Discriminant Analysis (LDA)	0.82	0.81	0.98	0.82	0.9
Quadratic Discriminant Analysis (QDA)	0.87	0.87	0.87	0.87	0.95
Proposed System	0.99	0.98	0.98	0.98	0.98

## Data Availability

All the necessary data and results are available within the manuscript, and the restricted data supporting this study’s findings are not available. The participants of this study did not give written consent for their data to be shared publicly, so supporting data is unavailable due to the sensitive nature of the research.
